# Anal infections with concomitant *Chlamydia trachomatis *genotypes among men who have sex with men in Amsterdam, the Netherlands

**DOI:** 10.1186/1471-2334-11-63

**Published:** 2011-03-14

**Authors:** Koen D Quint, Reinier JM Bom, Wim GV Quint, Sylvia M Bruisten, Maarten F Schim van der Loeff, Servaas A Morré, Henry JC de Vries

**Affiliations:** 1 DDL Diagnostic Laboratory, Voorburg, the Netherlands; 2 Department of Dermatology, Leiden University Medical Centre, Leiden, the Netherlands; 3 Public Health Laboratory, Cluster for Infectious Diseases, Public Health Service of Amsterdam (GGD Amsterdam), Amsterdam, The Netherlands; 4 Centre for Immunity and Infectious Diseases Amsterdam (CINIMA), Academic Medical Centre, University of Amsterdam, the Netherlands; 5 The Department of Pathology, Laboratory of Immunogenetics, VU University Medical Centre, Amsterdam, the Netherlands; 6 Department of Dermatology, Academic Medical Centre, University of Amsterdam, the Netherlands; 7 STI Outpatient Clinic, Cluster for Infectious Diseases, Public Health Service of Amsterdam (GGD Amsterdam), Amsterdam, the Netherlands

## Abstract

**Background:**

Lymphogranuloma venereum (LGV) proctitis is caused by *Chlamydia trachomatis *(Ct) genotype L and is endemic among men who have sex with men (MSM) in western society. Genotype L infections need to be distinguished from non-LGV (genotypes A-K) Ct infections since they require prolonged antibiotic treatment. For this purpose, an in-house developed *pmpH *based LGV polymerase chain reaction (PCR) test is used at the Amsterdam STI outpatient clinic. We investigated retrospectively the anal Ct genotype distribution, and the frequency of concomitant genotype infections in MSM infected with LGV and non-LGV Ct infections. To detect concomitant Ct genotype infections, the *pmpH *LGV PCR and genoTyping Reverse Hybridization Assay (Ct-DT RHA) were used.

**Methods:**

A total of 201 Ct positive rectal swabs from MSM were selected, which were previously diagnosed as either LGV (n = 99) or non-LGV Ct infection (n = 102) according to the algorithm of Ct detection by the commercially available Aptima Combo 2 assay followed by an in-house *pmpH *LGV PCR. The samples were retested with the commercially available Ct-DT RHA, which differentiates between 14 major genotypes and is able to detect concomitant Ct genotypes.

**Results:**

Excellent genotyping agreement was observed between the Ct-DT RHA and the *pmpH *LGV PCR (Kappa = 0.900, 95%CI = 0.845-0.955, McNemar's p = 1.000). A concomitant non-LGV genotype was detected in 6/99 (6.1%) LGV samples. No additional LGV infections were observed with the Ct-DT RHA among the non-LGV Ct group. In the non-LGV group genotype G/Ga (34.3%) was seen most frequent, followed by genotype D/Da (22.5%) and genotype J (13.7%). All LGV infections were caused by genotype L2.

**Conclusions:**

Concomitant non-LGV genotypes do not lead to missed LGV proctitis diagnosis. The *pmpH *LGV PCR displayed excellent agreement with the commercially available Ct-DT genotyping RHA test. The genotypes G/Ga, D/Da and J were the most frequent non-LGV Ct strains in MSM.

## Background

*Chlamydia trachomatis *(Ct) is the most common sexually transmitted bacterial disease worldwide. A Ct infection can infect different mucosal linings, with the majority of cases in the urogenital tract but also the rectum, oropharynx or conjunctiva.

In men who have sex with men (MSM), the rectum is often the only infected site, without a concurrent Ct infection in the urogenital tract [1]. Like urogenital infections, most rectal Ct infections remain asymptomatic [2]. Nevertheless, an asymptomatic rectal Ct infection can contribute to HIV transmission due to mucosal damage and recruitment of dendritic cells [3].

In general, Ct infections caused by non-LGV Ct genotypes (D-K) give few or no symptoms since they remain confined to the mucosal lining and do not trigger overt immunological reactions [4]. Ct infections caused by the genotypes L1-L3 give rise to an invasive, symptomatic and ulcerative infection called lymphogranuloma venereum (LGV). Since 2004 an ongoing epidemic of LGV proctitis is affecting MSM in Western countries, of which many are co-infected with HIV and hepatitis C [5]. It is highly recommended to differentiate an LGV Ct infection from a non-LGV Ct infection, since an LGV Ct infection requires longer antibiotic treatment [6]. Nowadays several assays are available to differentiate between an LGV genotype and a non-LGV genotype [7-11].

At the sexually transmitted infections (STI) outpatient clinic of the Public Health Service (MHS) of Amsterdam all MSM engaging in receptive anal sex in the previous 6 months are screened for anal Ct infections by the Aptima Combo 2 system (GEN-PROBE, San Diego, USA) and, if Ct positive, further tested with a *pmpH *based in-house developed real-time PCR to discriminate between an LGV genotype and a non-LGV genotype [7]. The MSM population visiting STI clinics are often diagnosed simultaneously with multiple STIs [12]. It has been suggested that in case of a mixed Ct infection with both LGV and non-LGV genotypes, a low bacterial load of the LGV genotype could be missed due to primer competition for different genotypes [13].

The genotyping step from the Ct-Detection and genoTyping (DT) assay (Labo Biomedical Products BV, Rijswijk, The Netherlands) can simultaneously genotype multiple Ct genotypes (A, B/Ba, C, D/Da, E, F, G/Ga, H, I/Ia, J, K, L1, L2/L2a, and L3) by a dual target PCR, targeting *Omp*A and the Ct endogenous plasmid, followed by a reverse hybridization assay (RHA) [8,14]. This RHA platform can detect concomitant infections, even if one genotype is present in a much lower concentration compared to additional genotypes (up to ratios of 1:1000) [15].

In the current study, we evaluated the diagnostic performance of the *pmpH *LGV PCR, used at the MHS of Amsterdam for the diagnosis of LGV infections, by retesting 100 LGV Ct positive and 100 non-LGV Ct positive samples with the Ct-DT RHA PCR system. In addition, we investigate the anal concomitant Ct genotype infections. Finally, we studied the non-LGV genotype distribution in rectal samples from MSM.

## Methods

### Clinical specimens

In the STI clinic of the Public Health Service of Amsterdam non-LGV Chlamydia and LGV infections in anal samples of MSM are diagnosed according to an algorithm consisting of Ct detection with the Aptima Combo 2 test, followed by differentiation with the *pmpH *LGV PCR, briefly described below. We selected samples from the archive (-80ºC freezer) of the Public Health Laboratory from a period starting in December 2009 and going back in time, until we had about 100 LGV samples and about 100 non-LGV Ct samples, as described before [16]. Since the prevalence of LGV Ct infections among clients of the STI clinic is much lower than the prevalence of non-LGV Ct infections, the period from which the LGV positive samples were selected was longer than the period from which the non-LGV samples were selected. Participants with non-LGV proctitis were treated with doxycycline 100 mg twice daily for a minimum of 7 days and those with LGV proctitis for a minimum of 21 days, directly after diagnosis. For this study we did not use any additional data or samples other than obtained in the routine screening procedure of the clinic. Therefore, neither additional ethical approval, nor additional patient consent was considered necessary. All samples were de-identified before starting the analyses. No history about the patient's STD and HIV status was available.

### Algorithm of Ct detection and LGV differentiation for rectal swabs from MSM visiting the STD clinic from the MHS

Rectal swabs from MSM were first tested for Ct with the commercially available Aptima Combo 2 Ct-RNA TMA assay, according to the manufacturer's instruction (GEN-PROBE, San Diego, USA). All Ct positive samples were further tested with the in-house *pmpH *LGV real time PCR, of which the primers and probes were described previously [7]. Briefly, the real time PCR was performed in 20 μL, containing Platinum Quantitative PCR SuperMix-UDG (Invitrogen, Breda, the Netherlands), 2 μL of isolated DNA, 4.3 mM MgCl_2_, 0.40 μM of primer F3 LGV, 0.39 μM of primer F4 non-LGV and 0.92 μM of primer R2 LGV/non-LGV, 0.15 μM of probe LGVtotP and 0.21 μM of probe P4 non-LGV. Cycling conditions for the real-time PCR were: uracil DNA glycosylase step at 50ºC for 2 minutes and denaturation at 95ºC for 2 minutes, followed by 45 cycles of 15 seconds at 95ºC and 1 minute at 60ºC. All tests were performed on a Rotor-Gene 6000 (Qiagen, Venlo, the Netherlands). Samples that were negative with the *pmpH *real time PCR were considered to be non-LGV Ct infection, since the sample was already determined Ct positive with the more sensitive Aptima Combo 2 assay.

### DNA isolation

Isolation of the DNA was performed at the MHS. DNA was isolated from 200 μl transport medium (GEN-PROBE, San Diego, USA) by adding 500 μl lysisbuffer (bioMérieux, Boxtel, the Netherlands), 1 μl glycogen (20 mg/mL, Roche Diagnostics, Almere, the Netherlands) and 700 μL isopropanol (-20ºC). The precipitate was washed twice with 70% ethanol and subsequently dissolved in 50 μl 10 mM Tris buffer (pH 8.0).

### Ct-DT RHA

The Ct-DT PCR and Ct-DT RHA were performed according to the manufacturer's instructions (Labo Biomedical Products BV, Rijswijk, The Netherlands) and as described previously [8,17]. No Ct-DT detection with a Ct-DNA enzyme immunoassay was performed between the amplification and genotyping step, since all samples were already previously determined as Ct positive by the Aptima Combo 2 assay.

*Ct-DT PCR: *A 10 μl aliquot of extracted DNA was used for each PCR reaction. The Ct PCR primer set was used to amplify all known genotypes available in GenBank http://www.ncbi.nlm.nih.gov/genbank. Briefly, this multiplex primer set amplifies a small fragment of 89 base pairs from the endogenous plasmid and a fragment of 160/157 base pairs from the Variable Region 2 of the *ompA *gene. The standard PCR program involves a 9-minute preheating step at 94ºC for AmpliTaq Gold activation, followed by 40 cycles of amplification (30 seconds at 94ºC, 45 seconds at 55ºC and 45 seconds at 72ºC) and a final 5-minute elongation at 72ºC.

*Ct-DT Reverse Hybridization Assay (RHA): *The Ct-DT RHA contained 19 probes for the endogenous plasmid, the Ct serogroups (B, C, and I) and the 14 genotypes (A, B/Ba, C, D/Da, E, F, G/Ga, H, I/Ia, J, K, L1, L2/L2a, and L3). Genovar L2b is detected as L2. The probe for the endogenous plasmid was added to increase sensitivity for Ct-detection, since genotyping on the plasmid is not possible. In short, 10 μl of the biotin-labeled PCR product was mixed with 10 μl of denaturation solution and incubated at 50°C for 1 hour, followed by several washing steps. All incubations and washing steps were performed automatically in an AutoLipa instrument (Tecan Austria GmbH, Salzburg, Austria).

### Statistical analysis

The level of agreement between the *pmpH *LGV PCR and the Ct-DT RHA was determined using Cohen's Kappa for four categories. A two-tailed McNemar's test was performed to investigate differences between both assays. The level of statistical significance was set at p < 0.05. All statistical analyses were performed in SPSS version 17.0 (SPSS version 17.0; Gorinchem, the Netherlands). Serovar distribution analysis was performed in each group separately, since the LGV and non-LGV Ct infections were obtained consecutively during different time frames.

## Results

### Agreement between the Ct-DT RHA and the *pmpH *LGV PCR

An excellent agreement was observed between the Ct-DT RHA and the *pmpH *LGV PCR in differentiating between an LGV and a non-LGV Ct infection (Kappa value = 0.900, 95% CI = 0.845 - 0.955, McNemar's p = 1.000) (Table 1). 189/201 (94%) samples showed diagnostic concordance between the two assays, consisting of 91 LGV infections, 82 non-LGV infections, 4 mixed LGV/non-LGV infections and 12 non-typable infections (yet 6/12 of these non-typable samples were still Ct plasmid positive with the Ct-DT RHA). A total of 12 (6%) discordant samples were observed. The discordant samples between both assays consisted of 2 infections that were diagnosed as LGV with the *pmpH *LGV PCR, but determined as a mixed LGV/non-LGV infection with the Ct-DT RHA and also 2 infections were determined as LGV infection with the Ct-DT RHA, but diagnosed as mixed LGV/non-LGV infection with the *pmpH *LGV PCR. Four Ct infections diagnosed as non-typable with the *pmpH *LGV PCR, were determined as a non-LGV Ct infection with the Ct-DT RHA, and vice versa 4 samples were non-typable (3 Ct endogenous plasmid Ct positive and 1 Ct negative) with the Ct-DT RHA but diagnosed as non-LGV Ct infection with the *pmpH *LGV PCR. All 99 LGV infections were confirmed with the Ct-DT RHA and no additional LGV infections were observed in the non-LGV group.

**Table 1:** Genovar differentiation results of the *pmpH *real time PCR and the Ct-DT RHA of 201 Aptima combo 2 *C. trachomatis *positive rectal swabs from men who have sex with men visiting the Amsterdam STI clinic between August 2008 and December 2009.

	*pmpH *LGV PCR				
	
Ct-DT RHA	LGV	Non-LGV	LGV+non-LGV	Non-typable*	Total
LGV	91	-	2	-	93
Non-LGV	-	82	-	4	86
LGV+non-LGV	2	-	4	-	6
Non-typable*	-	4	-	12	16
Total	93	86	6	16	201

Overall Kappa value = 0.900 (95% CI = 0.845 - 0.955), McNemar's p = 1.000

* The Ct-DT RHA non-typable samples consist of 9 Ct endogenous plasmid positive samples and 7 Ct negative samples. All 16 non-typable samples with the *pmpH *LGV PCR showed a negative result.

### Ct genotype distribution

The Ct-DT RHA was used to investigate the genotype distribution among the 201 rectal samples. All 99 LGV Ct infections consisted of genotype L2. Six of these 99 patients had a co-infection with a non-LGV Ct strain: D/Da (n = 2), E (n = 2), G (n = 1) and J (n = 1). Among the non-LGV Ct infections in MSM, genotype G/Ga (34.3%) was most prevalent, followed by genotype D/Da (22.5%) and genotype J (12.7%) (Table 2). One concomitant genotype infection was observed in the non-LGV group, containing the genotypes E&F. A trend toward significance was observed for concomitant infections with a LGV type compared to concomitant non-LGV genotype infections (6.1% vs. 1.0%, Fisher's exact p = 0.1244).

**Table 2:** Genotype distribution of the 102 non-LGV positive rectal swabs from MSM visiting the Amsterdam STI clinic from August 2008 to December 2009.

Diagnosis	non-LGV	
	**N**	**%**
	
**Single infections**		
Genotype D/Da	23	22.5
Genotype E	7	6.9
Genotype F	5	4.9
Genotype G/Ga	35	34.3
Genotype J	14	13.7
Genotype K	1	1.0
Non-typable	16	15.7
**Mixed infections**		
Genotypes E&F	1	1.0

Total	102	100

The genotype distribution was determined with the Ct-DT RHA system.

## Discussion

The Ct-DT RHA and the *pmpH *LGV PCR showed an excellent agreement in differentiating between an LGV and a non-LGV Ct infection. No additional LGV infections were observed with the Ct-DT RHA, indicating a good diagnostic performance of the *pmpH *LGV PCR for the detection of LGV infections. Still 2 concomitant non-LGV infections were missed with both assays among the LGV group. This discrepancy might be due to a lower bacterial load of the non-LGV Ct infection in the isolated DNA and/or primer competition during the PCR. However, this observation has no clinical relevance, since all patients received treatment for an LGV infection, which is more than sufficient for a non-LGV infection. It would be of more importance when concomitant LGV infections were missed, but fortunately this was not the case.

The current study showed that 1.0% of the Ct infections within the non-LGV group consisted of a concomitant non-LGV infection. This study also provides an estimate of the prevalence of non-LGV co-infections (6.1%) among MSM infected with LGV. The results of concomitant genotypes in both groups are comparable with previous studies of the urogenital tract in the general population, although the percentage in the non-LGV group seems slightly lower [18-22].

**Figure 1:**
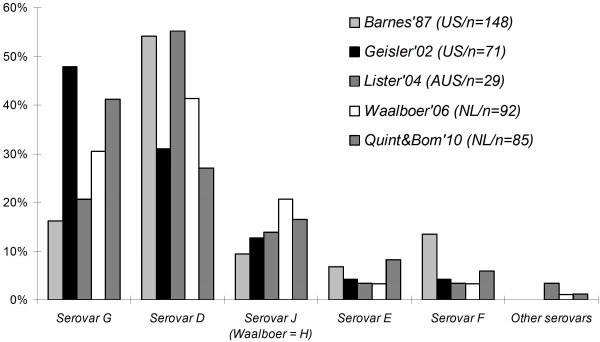
**Rectal non-LGV Ct genotype distribution in men who have sex with men: a comparison with previous studies **[23,24,27,28]. Non typable infections in the non-LGV group were excluded.

The Ct-DT RHA not only differentiates between an LGV and non-LGV Ct infection, but is also able to further differentiate between the 14 major Ct genotypes. The most frequently observed Ct genotypes were the genotypes G/Ga, D/Da and J in the non-LGV group. This distribution is similar to the distributions found in rectal samples from other MSM studies performed previously at different time periods (1987 to 2010) and different geographic locations (North America, Europe and Australia, Figure 1) [23-28]. Only one study (the Netherlands) showed discordant results, as genotype J was totally absent and a high number of genotype H was present [27]. In that study the RFLP technique was used to discriminate between the genotypes. As the RFLP patterns of genotype H and J are very similar, it is possible that in that study the J genotypes might have been mistaken for H genotypes.

The anal genotype distribution in MSM, dominated by types G, D and J, differs significantly from the distribution found in genital samples from the heterosexual population, where E, F, and D are the most frequently observed genotypes [14,29-31]. Apart from network associated factors, also tissue tropism could explain this difference in distribution, certainly since a recent study revealed an association between rectal tropism and polymorphisms of open reading frames within genotype G [32]. Also, on rectal swabs from heterosexual women an elevated prevalence of genotype G/Ga compared to urogenital samples was found, although this was not significant [33].

All LGV infections were caused by genotype L2. Previous sequencing identified the Ct genovariant L2b, which is a genovariant of genotype L2 [7]. This genotype is highly specific for LGV proctitis in the current epidemic among MSM, while during the 1980s-1990 s also genotype L1 was described among MSM [34].

## Conclusion

The Ct-DT RHA and the *pmpH *LGV PCR had an excellent agreement in differentiating LGV from non-LGV Ct infections. Also, concomitant non-LGV genotypes do not lead to missed LGV proctitis diagnosis with the *pmpH *LGV PCR. The anogenital genotype G/Ga, D/Da and J were the most frequent genotypes in rectal samples from MSM infected with a non-LGV strain. This genotype distribution differs from that of urogenital samples in the general population. All LGV infections were caused by genotype L2, which is in line with previous observations among MSM.

## Conflict of interests

The authors declare that they have no competing interests.

## Authors' contributions

KDQ and RJMB performed the DNA isolation, the Ct-DT RHA and the *pmpH *LGV PCR, managed the data and wrote the first draft of the manuscript. WGVQ and SMB both supervised the practical work and data management. MFSL validated the statistical analyses. SAM and  HJCV advised and developed the concept and selected the samples. All authors contributed to subsequent versions of the manuscript and saw and approved the final version.

## Pre-publication history

The pre-publication history for this paper can be accessed here:

http://www.biomedcentral.com/1471-2334/11/63/prepub
